# Comments: "Are smartphones a tool to cope with the fear of childbirth? The correlation between the fear of loss of connection and the fear of childbirth"

**DOI:** 10.1590/1806-9282.20260028

**Published:** 2026-08-10

**Authors:** Sami A. Gabr

**Affiliations:** 1 Mansoura University, Faculty of Medicine, Department of Medical Microbiology and Immunology, Genetics & Microbiology Infection Control Unit – Mansoura, Egypt.

## COMMENTARY

Smartphones have transcended their original purpose as communication tools, evolving into comprehensive portable ecosystems that seamlessly integrate information access, health monitoring, and social connection into daily routines. Their widespread use has facilitated the rapid sharing of health information, real-time tracking of symptoms, and remote interactions with healthcare providers, all of which enhance patient engagement and decision-making during pregnancy^1^.

In low- and middle-income regions, smartphones play a pivotal role by addressing challenges related to geographic barriers and limited healthcare infrastructure. They promote adherence to medical appointments, enable teleconsultations when in-person visits are not possible, and extend access to much-needed care^2^. As such, smartphones now serve as vital instruments for improving health literacy and ensuring the continuity of care.

The psychological impact of smartphone reliance warrants thoughtful consideration. While digital reassurance and social connectivity can help reduce uncertainty and provide empowerment for pregnant women, overuse may foster dependency, amplify anxiety during periods of disconnection, and disturb sleep patterns. These effects can indirectly shape obstetric behaviors and outcomes^3,4^. This dual-sided impact highlights the importance of promoting guided, clinically approved, and balanced use of digital tools, rather than allowing unrestricted or unregulated practices. In this regard, smartphone use during pregnancy may provide reassurance and support while also increasing the risk of dependency and anxiety when use of smartphones becomes excessive.

Nomophobia, or no-mobile-phone phobia, is increasingly recognized as a behavioral dependency characterized by feelings of anxiety, discomfort, or even panic when individuals lack access to their smartphones or digital connectivity. Instead of fitting the mold of a traditional specific phobia, nomophobia represents a pattern of reassurance-seeking behaviors, fueled by the fear of losing connection to information, social support, or control over one's surroundings^5^. This condition is particularly common among younger, digitally active populations and is linked to unhealthy coping mechanisms, compulsive device-checking habits, and heightened susceptibility to stress^6^. With smartphones now deeply integrated into daily routines, the line between practical use and dependency has become increasingly blurred.

Nomophobia is clinically relevant because it intersects with anxiety, sleep disruption, and impaired emotional regulation. Repeated device checking offers short-term relief yet reinforces dependency, creating a cycle in which the absence of connectivity heightens tension and perceived threat^7^. Studies also report associations between high smartphone dependence and poorer sleep quality, reduced attention, and increased psychological distress^4^. During pregnancy, a period already characterized by heightened vigilance and uncertainty, these dynamics may intensify, leading women to rely on smartphones as primary tools for reassurance, while paradoxically sustaining anxiety when connectivity is threatened^4,6^.

Pregnant women often use smartphones to access health information, track fetal development, communicate with peers and healthcare providers, and receive lifestyle guidance. Mobile health (m-health) applications have the potential to enhance participation in antenatal care, improve health literacy, and aid maternal decision-making when they are clinically approved and appropriately guided^1,8^. However, unguided or excessive reliance carries risks: exposure to unverified content, heightened fear, increased screen time, and reinforcement of nomophobia tendencies^9^.

However, excessive or unguided reliance on digital tools may increase exposure to unverified information and reinforce reassurance-seeking behaviors associated with nomophobia^9^. Therefore, it's important that these digital tools should complement, rather than replace, professional antenatal counseling and psychosocial support. These findings of this study explore how individual differences, such as attachment styles, may influence the relationship between smartphone use and reassurance-seeking behaviors. Additionally, support the importance of smartphones as supportive educational tools and as sources of maladaptive reassurance-seeking behaviors.

**Figure 1:**
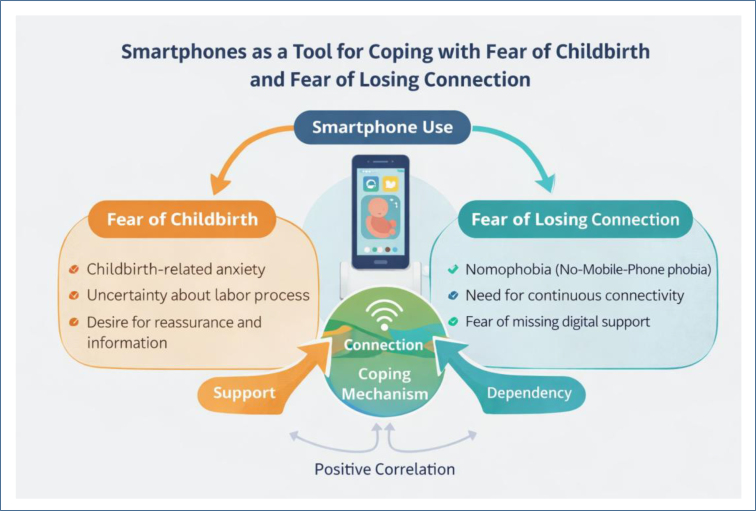
Illustrates the reinforcing loop that may link uncertainty, digital checking, and heightened childbirth fear.

In addition, an important association between smartphone dependence (nomophobia) and fear of childbirth among primiparous women was highlighted, demonstrating that approximately one in five participants exhibited both extreme nomophobia and clinical fear of childbirth. This large cross-sectional sample (n=3,870), use of validated psychometric tools, and multivariate modeling contribute substantially to the literature on digital behavior in pregnancy^10^. Notably, daily smartphone use correlated with increased fear of childbirth, while fetal-development applications were associated with modestly lower fear levels^10^. These findings highlight smartphones as bidirectional tools (Figure 1): they may educate and reassure, yet also foster digital dependence when used for repetitive reassurance-seeking.

Figure 1 illustrates the proposed reinforcing feedback loop linking fear of childbirth, reassurance-seeking behaviors, smartphone dependence, and nomophobia. The figure conceptually demonstrates how pregnancy-related anxiety and uncertainty may encourage repeated smartphone checking to obtain reassurance through health information, fetal-development tracking, or online social support. Although this behavior may provide temporary emotional relief, repetitive digital reassurance-seeking may gradually reinforce dependence on continuous connectivity and increase distress when smartphone access is limited. The model, therefore, highlights the potential bidirectional interaction between pre-existing anxiety traits and problematic smartphone use during pregnancy rather than suggesting a strictly unidirectional causal relationship.

Although Bingol et al. demonstrated a significant association between nomophobia and childbirth fear, the cross-sectional nature of the study does not clarify causality. It is plausible that women with higher baseline anxiety, uncertainty intolerance, or heightened vigilance toward pregnancy-related risks are more inclined to engage in excessive smartphone use as a coping mechanism. In this context, smartphones may function as easily accessible tools for reassurance-seeking, allowing repeated checking of symptoms, fetal development information, or online peer experiences. However, this coping behavior may paradoxically maintain or intensify anxiety by increasing exposure to alarming or contradictory health information and reinforcing dependency on continuous digital connectivity^7,10^.

A reinforcing feedback loop may therefore exist between reassurance-seeking behaviors and nomophobia during pregnancy. Repeated smartphone checking can provide temporary emotional relief, but this short-term reduction in anxiety may strengthen compulsive reliance on the device over time. Consequently, periods of disconnection or inability to access digital information may trigger heightened distress, uncertainty, and fear of losing control. Similar behavioral reinforcement patterns have been described in studies linking problematic smartphone use with anxiety, fear of missing out, and impaired emotional regulation^3,7^. During pregnancy, when emotional sensitivity and health-related concerns are naturally elevated, this cycle may become even more pronounced, particularly among primiparous women who often experience greater uncertainty regarding childbirth outcomes.

Importantly, these observations suggest that smartphones should not be viewed solely as harmful or beneficial tools, but rather as modifiers of existing psychological vulnerability. It was reported that increasing nomophobia levels in pregnant women were positively associated with higher anxiety, stress, and depression scores^11^. In addition, examined smartphone-use behaviors among pregnant women highlighted the psychological and behavioral implications of excessive smartphone reliance during pregnancy^12^. Women with pre-existing anxiety traits may derive reassurance and support from digital health applications when use is balanced and guided by healthcare professionals. In contrast, excessive or unguided use may amplify nomophobia and sustain fear-related cognitions^11,12^.

This interpretation strengthens the unique contribution of the present commentary by framing smartphone dependence and fear of childbirth within a dynamic behavioral model rather than a simple unidirectional association. Such a perspective also supports the need for longitudinal studies examining whether anxiety precedes problematic smartphone reliance, whether smartphone dependency exacerbates childbirth fear, or whether both mechanisms interact simultaneously within a self-reinforcing psychological cycle.

The results are consistent with previous evidence showing that many pregnancy applications vary widely in clinical quality, accuracy, and privacy safeguards^9,13^, while carefully designed and monitored digital interventions can improve diet and gestational weight control in selected populations^14^. Concurrently, nomophobia has been increasingly recognized as a behavioral health concern, particularly among younger users^3,15^. Together, the literature indicates that technology is neither inherently protective nor harmful—its impact depends on content integrity, usage patterns, and integration with professional care.

Smartphone use is playing an increasingly important role in maternal psychology by influencing how pregnant women seek information, manage uncertainty, and regulate their emotions. Access to updates on fetal development, online communities of peers, and digital health tools can help women feel more in control and connected, ultimately reducing anxiety for some. However, constantly seeking reassurance, being exposed to conflicting or alarming content online, and fearing disconnection can increase vigilance and stress, especially for women with existing anxiety issues. These two psychological effects suggest that smartphones not only serve as sources of information but also as emotional amplifiers, capable of either supporting or undermining coping strategies depending on the quality of content, frequency of use, and guidance from healthcare professionals^7^.

**Figure 2:**
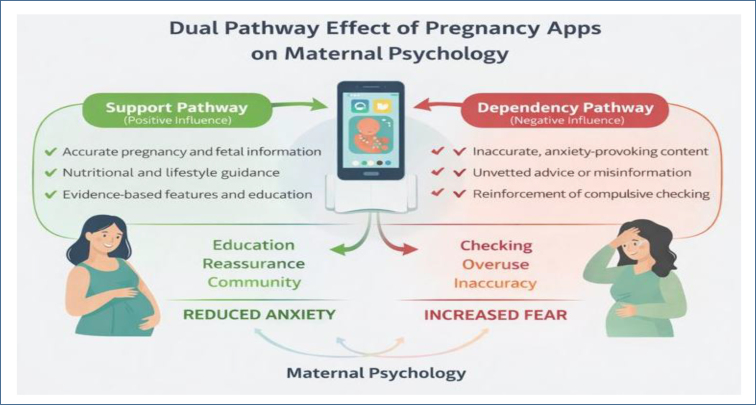
Dual pathway model of pregnancy application use in smartphones and maternal psychology.

Figure 2 summarizes the two major psychological pathways associated with pregnancy smartphone applications. On one side, clinically reliable applications provide structured education, fetal-development tracking, lifestyle support, and access to credible guidance, which may enhance health literacy, perceived control, preparedness, and maternal confidence, thereby helping to reduce fear of childbirth. On the other side, excessive reassurance-seeking, frequent checking behaviors, exposure to unverified information, and reliance on continuous digital connectivity may reinforce nomophobia and increase anxiety. Therefore, the psychological impact of smartphone applications during pregnancy depends largely on usage patterns, content quality, and the presence of appropriate professional guidance. As reported in Figures 1 and 2, the psychological impact of smartphone applications during pregnancy depends largely on how these tools are used and integrated into antenatal care.

This dependence may heighten anxiety when the phone is unavailable, increase rumination, and occasionally escalate worry when contradictory or alarmist information appears online. Thus, the same digital tool can both support coping and sustain psychological vulnerability, depending on the content quality, frequency of use, and presence of professional guidance.

This interpretation is further supported by recent evidence linking problematic smartphone dependence during pregnancy with broader dimensions of perinatal mental health. A recent study by Aker et al.^11^, demonstrated that increasing nomophobia levels in pregnant women were positively associated with anxiety, stress, and depressive symptoms, suggesting that problematic digital reliance may represent part of a broader psychosocial vulnerability profile during pregnancy. Similarly, recent research examining nomophobia and health literacy during pregnancy reported that excessive smartphone dependence may coexist with altered patterns of digital health information seeking, emphasizing the importance of guided and psychologically informed digital engagement during antenatal care.

These findings reinforce the growing recognition that perinatal mental health is closely influenced by digital behaviors, particularly among women who rely heavily on smartphones for reassurance, symptom monitoring, and emotional regulation. Consequently, integrating mental health screening, digital-use assessment, and evidence-based counseling into routine antenatal care may help identify vulnerable women early and reduce maladaptive reassurance-seeking behaviors associated with nomophobia and childbirth-related anxiety^11,16^. The studies showed that electronic screen exposure, including screen viewing time, smartphone addiction, and problematic smartphone use was associated with depression among women in early pregnancy^16,17^. Additionally, it was demonstrated that smartphone overuse, nighttime smartphone use, and social-networking–focused smartphone behaviors were independently associated with antenatal anxiety, supporting the importance of integrating digital behavior monitoring and mental health screening into prenatal care^17,18^.

Healthcare professionals involved in antenatal care may benefit from recognizing behavioral indicators of problematic digital reliance among pregnant women. Excessive reassurance-seeking through repeated symptom searches, compulsive checking of pregnancy applications, heightened distress when disconnected from smartphones, sleep disruption related to nighttime device use, and persistent anxiety triggered by online health information may all represent clinically relevant warning signs. Because fear of childbirth and nomophobia may coexist with underlying anxiety vulnerability, clinicians should consider incorporating brief psychosocial screening questions related to digital behaviors during routine antenatal visits. Early identification of maladaptive smartphone dependence may help prevent reinforcement of anxiety-related coping patterns and improve emotional well-being during pregnancy^3,7,10^.

Integrating these concepts into antenatal care requires a balanced and nonjudgmental approach that acknowledges both the benefits and risks of digital health engagement. Rather than discouraging smartphone use entirely, clinicians can guide pregnant women toward evidence-based applications, encourage scheduled rather than compulsive information-seeking behaviors, and discuss strategies for maintaining healthy digital boundaries, particularly before sleep or during periods of heightened anxiety. Counseling approaches may also include reassurance regarding normal pregnancy-related uncertainty, promoting face-to-face communication with healthcare providers, and encouraging participation in structured childbirth education programs. Such interventions may help transform smartphone use from a source of anxiety reinforcement into a supportive adjunct to comprehensive maternal care^1,8,10^. Digital health interventions can significantly reduce depressive and anxiety symptoms among perinatal women and highlight the growing role of smartphone-based mental health and improving emotional support during pregnancy and the postpartum period^18,19^.

Clinically, the findings support a balanced approach to smartphone use during pregnancy rather than discouraging digital engagement altogether. Healthcare providers should screen for fear of childbirth and problematic smartphone dependence, recommend evidence-based applications, and encourage healthy digital habits alongside antenatal education and psychosocial support. In parallel, application developers should incorporate features that reduce compulsive checking behaviors and promote mental health awareness^1,3,7,10,11,20^.

In conclusion, the study highlighted the complex relationship between smartphone dependence and fear of childbirth. Smartphones can be valuable tools for education, reassurance, and accessing maternal health support. However, excessive or unguided reliance on smartphones may also reinforce anxiety, reassurance-seeking behaviors, and nomophobia in vulnerable pregnant women. These findings underscore the importance of integrating digital technologies into antenatal care in a balanced and clinically supervised manner. It is crucial to ensure that digital support complements rather than replaces professional counseling and psychosocial care. Future research should utilize longitudinal, randomized, and mixed-methods designs to clarify causal relationships between smartphone use, anxiety traits, and fear of childbirth. Additionally, research should aim to distinguish between adaptive and maladaptive patterns of digital behavior across culturally diverse populations.

## Data Availability

The datasets generated and/or analyzed during the current study are available from the corresponding author upon reasonable request.
